# Innate Immune Response to Viral Infections at the Maternal-Fetal Interface in Human Pregnancy

**DOI:** 10.3389/fmed.2021.674645

**Published:** 2021-07-22

**Authors:** Ana Espino, Hicham El Costa, Julie Tabiasco, Reem Al-Daccak, Nabila Jabrane-Ferrat

**Affiliations:** ^1^Infinity, Université de Toulouse - CNRS – Inserm, CHU Purpan, Toulouse, France; ^2^Inserm UMRS976 - Université de Paris - Hôpital Saint-Louis, Paris, France

**Keywords:** pregnancy, viral infection, maternal-fetal transmission, innate immunity, Natural Killer cells, macrophages, cytokine environment

## Abstract

The placenta, the first and largest organ to develop after conception, not only nurtures and promotes the development of the conceptus, but, it also functions as a barrier against invading pathogens. Early phases of pregnancy are associated with expansion of specific subsets of Natural Killer cells (dNK) and macrophages (dMφ) at the maternal uterine mucosa, the basal decidua. In concert with cells of fetal origin, dNK cells, and dMφ orchestrate all steps of placenta and fetus development, and provide the first line of defense to limit vertical transmission. However, some pathogens that infect the mother can overcome this protective barrier and jeopardize the fetus health. In this review, we will discuss how members of the classical TORCH family (Toxoplasma, Other, Rubella, Cytomegalovirus, and Herpes simplex virus) and some emerging viruses (Hepatitis E virus, Zika virus, and SARS-CoV2) can afford access to the placental fortress. We will also discuss how changes in the intrauterine environment as a consequence of maternal immune cell activation contribute to placental diseases and devastating pregnancy outcomes.

## Introduction

Human pregnancy is associated with tremendous changes of the maternal uterine mucosa (endometrium), called decidua, to promote the implantation, and the development of the blastocyst. The interstitial implantation, in humans and non-human primates, is characterized by complete enclosure of the blastocyst by the decidua (1). A privileged crosstalk between the endometrium and a competent 256-cell blastocyst must be completed in a limited time called the implantation window. The outer surface of the blastocyst also is composed of cytotrophoblasts (CTBs), a unique cell type of the placenta, which will follow two differentiation programs (2). In the first program CTBs fuse to form the multinucleated syncytiotrophoblast (STB) outermost layer surrounding the inner core of the placental villi. The villous core containing mesenchymal/fibroblastic stroma stem cells, immature fetal capillaries and a population of yolk sac-derived macrophages termed Hofbauer cells (HBCs), is surrounded by a bilayer of CTBs. In the second program, CTBs of the cell column differentiate into interstitial and endovascular invasive cytotrophoblast cells (iCTBs). Subsequently, iCTBs invade the decidua and remodel the maternal spiral arteries through active replacement of smooth muscle and vascular endothelial cells. Completion of vascular remodeling by trophoblast cells which happens by the end of first trimester of pregnancy (10–12 weeks), promotes the switch from high-resistance to low-resistance vessels with enlarged lumen to ensure routing of high blood flow into the intervillous space (3). As the development advances, the highly branched villous trees of the placenta are bathed with maternal blood till parturition. The invasion fetal trophoblast cells and tissue remodeling are under strict control by maternal immune cells to ensure confinement of the placenta to the allocated space within time constraints of pregnancy. Flaws in these developmental steps have been clearly incriminated in many great obstetrical syndromes including pre-eclampsia and fetal growth restriction (FGR), both associated with faulty placental development (3).

Unlike the fetus, the placenta is in direct contact with maternal cells. Intermingling between fetal and maternal cells, which is mandatory for placenta and fetal development, happens within privileged sites (maternal-fetal interface, composed of the basal decidua, and the intervillous space). Hormones are able to prime the decidua-associated remodeling (2), complete “physiological change” requires the presence of trophoblast cells. The extent of trophoblast cell invasion and tissue remodeling, both necessary for efficient and healthy placenta in late pregnancy, are dictated by a finely tuned crosstalk between fetal cells and maternal tissue resident immune cells. Leukocytes represent more than 40% of total cells in the basal decidua (4). While a large fraction of immune cells are tissue resident leukocytes, many factors produced by decidual stromal cells and trophoblast cells could contribute to the recruitment of additional leukocytes from the periphery (5, 6). A high percentage of decidual immune cells are non-cytotoxic CD56 super bright Natural Killer (dNK) cells that lack the expression of the Fc gamma receptor IIIA, CD16, and are endowed with angiogenic and regulatory activities. In the first trimester of pregnancy, dNK cell population represents 70% of leukocytes (7–9). Their rate remains relatively high in the second trimester but declines in the third trimester to reach normal uterine level at parturition (10). Other immune cell populations are also present in first trimester basal decidua, although, at lower levels. Decidual macrophages (dMφ) represent 20–25% of total leukocytes, while dendritic cells and mast cells only represent 1–2% (11). The decidua contains only 5–10% T lymphocytes, including CD8, CD4, and γδT cells, but B cells are barely present. The low level of B cells suggests that it is very unlikely that any eventual antibody response would harm invading trophoblast cells (12). Minor immune cell subsets include innate lymphoid cells (ILCs) (13) and mucosal-associated invariant T cells (MAIT) (14). The interaction of these immune cells with decidual stroma and invading fetal trophoblast cells is crucial for the establishment of an environment rich in soluble mediators including hormones, growth factors cytokines, chemokines and lipids, which are necessary for the development of the placenta and of the fetus. Given their dominance and active contribution to protection against pathogens, this review will mainly discuss the key features of dNK cells and dMφ during healthy pregnancy as well as their functional adaptations during viral invasion, and how viral infections disrupt the maternal-fetal barrier resulting in congenital infections.

## Decidual NK Cells

Natural killer (NK) cells are innate lymphoid cells with documented anti-viral and anti-tumor functions. They are found in the bloodstream (pNK), where they represent up to 15% of circulating lymphocytes, but also in various tissues such as lymphoid organs, liver and uterus. NK cells are classified in two major populations according to the cell surface expression of CD56 (NCAM) and CD16. The majority of pNK cells (~95%) are CD56^dim^CD16^pos^ and characterized by a highly cytotoxic capacity, the remaining 5% are CD56^bright^CD16^neg^ (8, 15). At the implantation bed, the majority of dNK cells are CD56 super bright (dNK^bright^) that share some phenotypic similarities with the minor population of pNK and only a small fraction CD56^dim^ (dNK^dim^) (8, 15, 16). dNK cells express most of the activating and inhibitory NK cell receptors including the NKp30, NKp46, NKG2C, NKG2E, NKG2D, NKG2A, 2B4, CD85/LILRB1/ILT2 (LILRB1), and Killer-cell immunoglobulin-like family of receptors (KIR) such as KIR2DL1, KIR2DL2/3, KIR2DL4, KIR3DL2 (10, 16–18). However, the function of several NKR is different as compared with pNK cells (15, 19). For example, the inhibitory LILRB1 in the periphery is an activating receptor in the decidua, whist the co-activating 2B4 receptor functions as inhibitory receptors in dNK cells. dNK cells also express many other receptors that are not expressed on resting pNK cells. dNK cells have a constitutive expression of CD69 and CD25, and display differential expression of NKp44/NCR2 and NKp30/NCR3 splice variants implying the involvement of the decidual environment in shaping their cell fate (20). dNK cells also express high levels of integrins (αD, αX, β1, and β5) and other receptors such as CD53, CD63, and CD151 (8) as well as tissue residency markers (CD49a, CD9, and CD103). In addition, several chemokine receptors (CXCR3, CXCR4, CCR1, and CCR9) endow them with high mobility to warrant a fine-tuned dialogue with other cells (21).

dNK cells can be classified under three major subsets dNK1, dNK2, and dNK3. dNK1 is the most abundant subset (>30%), followed by dNK2 and dNK3 (22, 23). While all three subsets express of NKG2A, LILRB1 expression is a major hallmark of dNK1 cells. dNK1 cells also express the regulatory CD39 ATPase, involved in the shift between pro-inflammatory and immunosuppressive environment, large amounts of cytotoxic granules enriched in perforin and granzymes (8), and a combination of KIRs expression that further discriminates four dNK1 subgroups. Similar to dNK1, dNK2 cells express the NKG2C and NKG2E activating receptors as well as the NKG2A inhibitory receptor counterpart that recognize HLA molecules on fetal trophoblast cells. In addition, dNK2 cells express the ANXA1 and ITGB2 receptors. Finally, the third subset expresses CD160, CD161, TIGIT, KLRB1, ITGB2, and CD103 (22, 24). The differential levels of NKp44 and NKG2A receptors suggest the presence of at least two subgroups of dNK3 cells.

**Figure 1:**
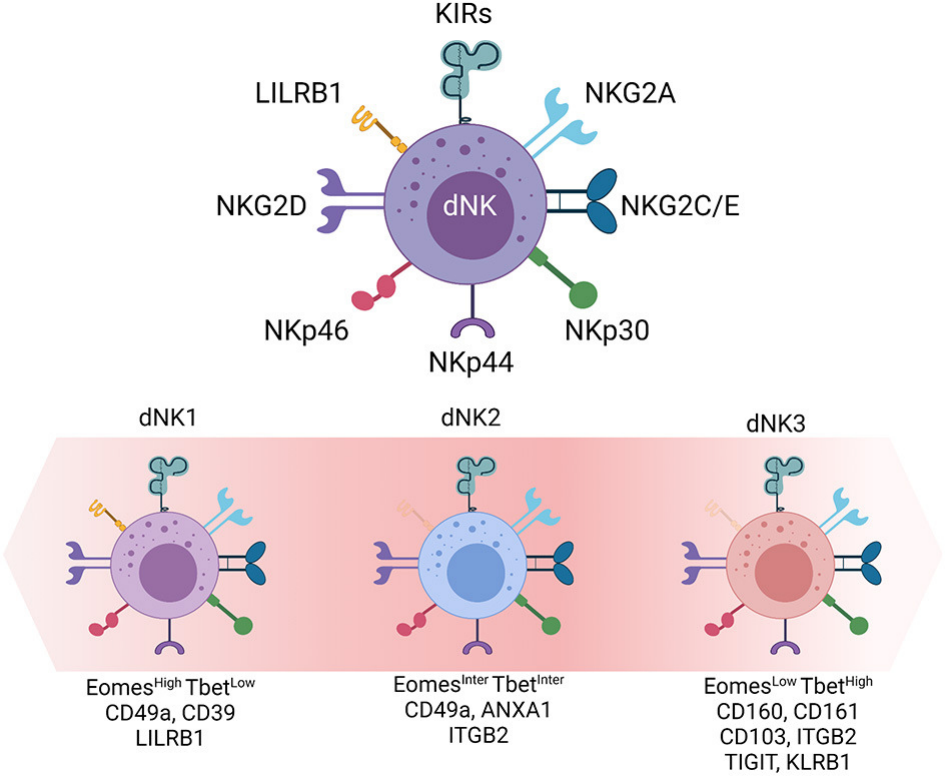
Decidual Natural Killer cells. dNK cells, the main innate immune cell population at the maternal-fetal interface. Inhibitory and activating receptors are presented. Transcription factors (Eomes and Tbet) and cell surface markers (LILRB1, ITGB2, CD160, CD161) defined by single cell RNA sequencing and large scale phenotyping allow the distinction of three dNK cell subsets (dNK1, dNK2, and dNK3).

In contrast to other tissues, all three CD49a^pos^ dNK cell subsets express the Eomes and Tbet transcription factors although, at variable levels. Compared to Eomes^high^Tbet^low^ dNK1 cells, dNK3 cells are Eomes^low^Tbet^high^ whilst dNK2 cell express intermediate levels of both transcription factors (Figure 1).

How these three subsets interact with each other's, with fetal trophoblast cells and decidual stromal cells (DSC), and whether they are all needed to direct the invasion of trophoblast cells, remodeling of the maternal-fetal interface and the development of the placenta are still in debate. Nonetheless, a finely tuned dialogue between different subsets of dNK cells, decidual cells, and invading trophoblast cells seems necessary to promote the complete establishment of the maternal-fetal interface but also the development of the fetus and the placenta (25).

Indeed, through direct interaction with iCTBs and production of a highly diverse secretome rich in cytokines, chemokines metalloproteinases, and growth promoting factors such as VEGF, IGF2, pleiotrophin, osteoglycin, and osteopontin), dNK cells direct the extent of trophoblast invasion, tissue remodeling as well as placental and fetal growth (26, 27). Some of these soluble factors are also important for the establishment of a local pro- and anti-inflammatory cytokine balance that is necessary for fetal tolerance as well as protection against mother-to-child transmission of pathogens.

By contrast to pNK, dNK cells are devoid of cytotoxicity during healthy pregnancies preventing harmful reaction against the semi-allogeneic trophoblast cells (15, 28). Several mechanisms have been suggested for their involvement in restricting dNK cell lytic machinery. These include (i) a defective polarization of the microtubule organizing center (MTOC), resulting in the formation of an immature immunologic synapse with target cells (29); (ii) the lack of SAP adapter molecule expression preventing activating signal from 2B4 (CD244) (19); (iii) the engagement of the NKG2A inhibitory receptor by its natural ligand HLA-E on trophoblast cells (30); and (iv) the expression of inhibitory isoforms of the Natural Cytotoxicity Receptors, NKp44 and NKp30 (16, 17, 20). Finally, the pressure of the local environment maintains cell cytotoxicity under a tight control (24).

Despite these regulatory mechanisms, dNK cell cytotoxic machinery can be unleashed resulting in failure in the harmonious mother-fetus dialogue and adverse pregnancy outcomes. For instance, patients with recurrent spontaneous abortion exhibit a change in the distribution of dNK cell subsets was associated with enhanced production of inflammatory cytokines and lytic granules, but impaired ability to produce growth promoting factors (27, 31). Thus, changes in dNK cell functions and the finely-tuned dialogue at the maternal-fetal interface is probably responsible for fetal trophoblast cells apoptosis and the resulting miscarriage. In addition, genetic studies demonstrated that particular combinations of HLA-C haplotypes and maternal inhibitory KIRs can be linked to defaults in trophoblast invasion and pregnancy disorders including pre-eclampsia pathologies (32, 33).

## Decidual Macrophages

Decidual macrophages (dMφ) represent the second largest population of leukocytes. The local environment at the maternal-fetal interface orchestrates dMφ differentiation and functions. In early stages of pregnancy, dMφ have an M1 phenotype, then they switch to a mixed M1/M2 profile during trophoblast cell invasion. Once the placenta is fully developed, they switch toward an M2 phenotype to prevent fetal rejection (34). Consequently, dMφ share features of pro-inflammatory and tolerogenic macrophages, simultaneously expressing M1 (CD64, CD80, CD86) and M2 markers (CD163, CD206, DC-SIGN) (35–39).

Similar to dNK cells, dMφ secrete a large panel of pro-angiogenic factors and pro- and anti-inflammatory cytokines (40, 41). Their localization in the vicinity of spiral arteries and secretion of metalloproteinases allude to their active involvement in vascular remodeling (42). In concert with dNK cells, dMφ promote trophoblast cell invasion and placental development (43, 44) while contributing to defense against threatening pathogens.

## Viral Infections During Pregnancy

**Figure 2:**
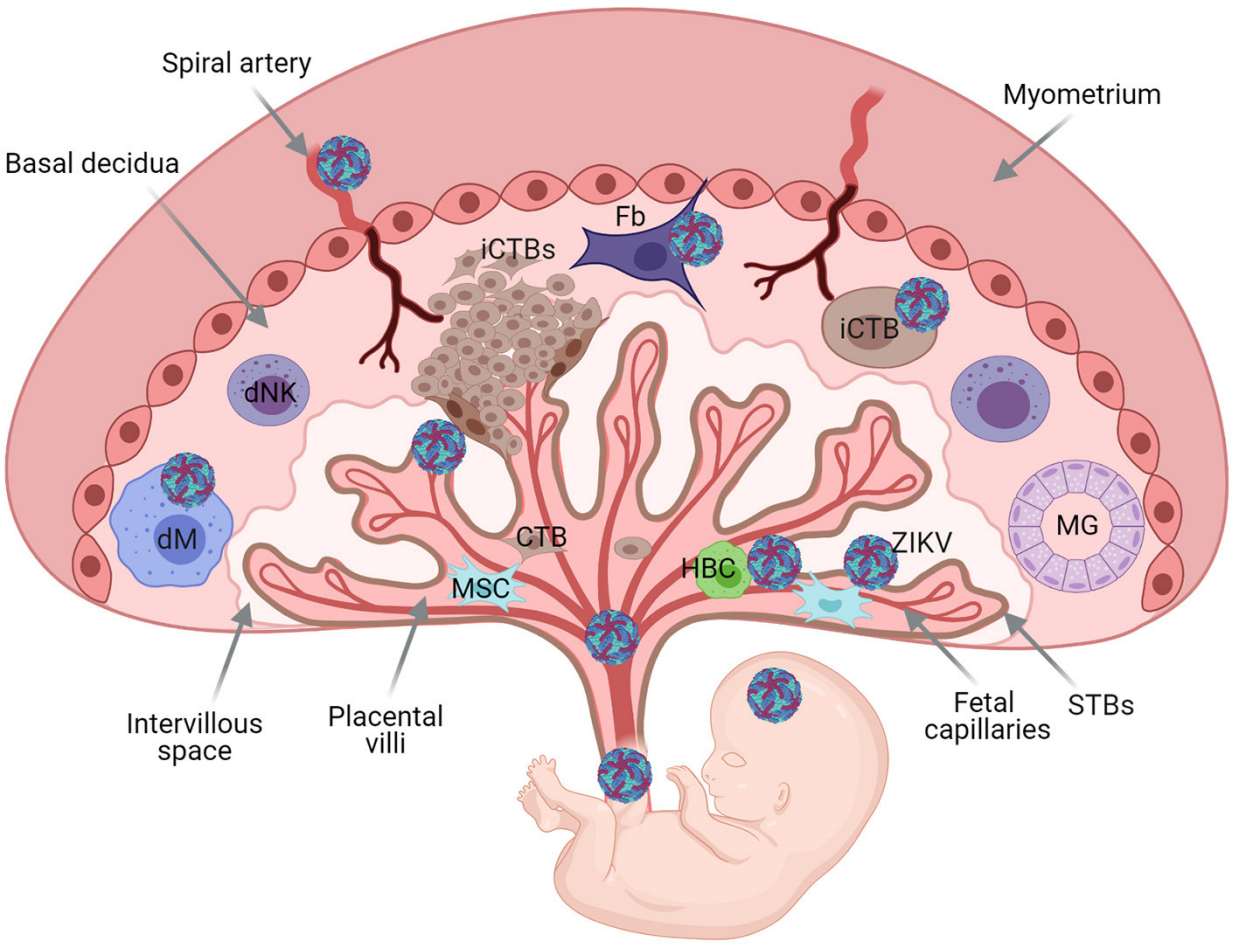
Schematic representation of the human maternal-fetal interface and fetus, and transmission of pathogens. Basal decidua containing decidual Natural Killer cells (dNK), maternal macrophages (dMφ), and stroma cells [mesenchymal (Ms) and fibroblasts (Fb)]. Maternal gland (MG). Extravillous invasive cytotrophoblast cells (iCTBs) invade the maternal decidua. Trophoblast Villous core contains trophoblast cells, mesynchymal cells, Hoffbauer cells (HBC), and fetal capillaries protected with an outermost layer of multinucleacted syncytiotrophoblast cells (STBs). Placental chorionic villi floating bathed by maternal blood within the intervillous space. Viruses can reach the fetus through hematogenous spread or through the basal decidua. Illustration of Zika virus (ZIKV), as model. ZIKV can replicate in the decidua and placental cells before dissemination to the fetus.

The maternal-fetal interface constitutes a physical and immune barrier shielding the fetus from pathogens in the maternal circulation. However, some pathogens especially viruses can reach the fetal compartment either through hematogeneous or decidual spread, resulting in congenital syndrome and high case-fatality (Figure 2). Even in the absence of maternal-to-fetal transmission, previous epidemics of many emerging viral threats have resulted in poor pregnancy outcomes including maternal and fetal morbidity and mortality and peripartum infections with severe sequelae.

### Threatening TORCH Pathogens During Human Pregnancy

The TORCH family [Toxoplasma, Other (syphilis, varicella-zoster, parvovirus B19), Rubella, Cytomegalovirus and Herpes simplex viruses 1 and 2] are well-recognized as pregnancy threating pathogens for human pregnancy.

The Human cytomegalovirus (HCMV), a large DNA virus that belongs to the herpesviridae family, is the most common cause of maternal and fetal morbidity and mortality. Primary maternal infection carries a 30–40% risk of vertical transmission. Congenital infection during the first trimester of pregnancy can result in severe congenital syndrome characterized by irreversible damage including deafness, psychomotor delays, myopathies, chorioretinitis, and devastating microcephaly (45). Even asymptomatic newborns can develop late neuro-developmental damage within the first 3 years of life (46). These congenital disorders could be partially attributed to severe placenta disruption with increased edema, massive inflammation, and avascular villi. HCMV infected placenta are characterized by massive viral-induced cytopathic effects in the villous capillaries and stromal cells and chronic villitis due to maternal inflammatory response.

The highly pathogenic Human immunodeficiency virus 1 (HIV-1) is an RNA virus from the retroviridae family. Histological analyses of first trimester placenta from untreated HIV-infected women revealed the presence of decidual necrosis and chorioamnionitis associated with the presence of HIV-1 in fetal tissues. Syncytin, the envelope glycoprotein of human endogenous retrovirus highly expressed by fetal trophoblast, mediates HIV entry through cell-to-cell fusion contributing to viral dissemination, and establishment of viral reservoir in deep tissues (47). Despite the high permissivity of trophoblast cells and dMφ, intrauterine transmission of HIV-1 is very rare during the first trimester of pregnancy (48) but occurs more frequently during labor and delivery. Although, ART can lower considerably the transmission rate, cesarean delivery is recommended for HIV-infected women with high viral loads.

### Emerging Viruses

Lessons from recent outbreaks and the current pandemic crisis, designated genotype 1 of Hepatitis E virus (HEV-1), Zika virus (ZIKV), and SARS-CoV2 as emerging viruses that can threaten human pregnancy.

Hepatitis E virus (HEV), a water-borne pathogen transmitted by the fecal-oral route, belongs to the *Hepeviridae* family. Strains that infect humans are classified in two species, *Orthohepevirus A* (eight genotypes) and *Orthohepevirus C* (49). HEV-1, prevalent in developing countries, is highly pathogenic during pregnancy with high maternal and fetal mortality rate (20%) due to fulminant hepatitis, hemorrhage, preeclampsia like syndrome, and severe placental diseases (50). The transmission rate of HEV-1 ranges from 50 to 100% (51). Trans-placental transmission has been documented by the presence of progeny virions in the fetal placenta and umbilical cord (52). Significant effort has been recently made by our team to understand mechanisms underlying HEV-1-related placental disorders (53). We demonstrate that pathogenic HEV-1 replicates efficiently both in maternal and fetal tissues, produces infectious progeny virions, and induces alteration of the local secretome leading to severe tissue damage.

ZIKV, a mosquito-borne *Flavivirus*, is able to surmount the physical and immunological barrier of the placenta to reach the developing conceptus in ~1 out of 10 infected pregnancies. In the 2015 outbreak in the western hemisphere, ZIKV was declared a serious public health concern, in particular for pregnant women due to surge of devastating microcephaly in newborns and increased frequency of placental pathology including preeclampsia, miscarriage and still birth (54, 55). Histological analysis of term placenta from congenital infected neonates, revealed the presence of immature villi with a large increase in the number of proliferating HBCs. Our group, among others, provided the proof that ZIKV targets a wide range of maternal and fetal cells including dMφ and HBCs, triggers massive inflammation and tissue damage, which may lead to placental insufficiency (56, 57). More recently, we described potential mechanisms for ZIKV-induced pathogenesis during early pregnancy (58). We showed that the virus usurps the placenta lipidome and the mitochondrial network to build replication factories necessary for the production of infectious progeny virions, and to overcome the placental intrinsic defense mechanisms through impairment of the homeostatic equilibrium of pro-/anti-inflammatory environment.

Pregnancy increases the risk of adverse maternal and offspring outcomes from many respiratory viral infections including Influenza virus. In December 2019, Wuhan, China, was faced with the outbreak of the deadliest acute respiratory distress syndrome, COVID-19 disease. On March 11, 2020 COVID-19 was declared a pandemic by the World Health Organization. The etiological agent of COVID-19, SARS-CoV2, is a newly identified member of a zoonotic family of coronaviruses responsible for severe acute respiratory syndrome (SARS). The former two family members SARS-CoV and Middle East respiratory syndrome (MERS)-CoV are the respective etiological agents responsible for the 2002 and 2012 outbreaks (59, 60).

Although, the COVID-19 pandemic has now infected large numbers of pregnant women, the risks and specific effects of SARS–CoV-2 are still largely unknown. Early reports of Chinese pregnancy cohorts did not provide evidence for maternal-fetal transmission in late gestation, but infection was clearly associated with increased risk of admission to intensive care, miscarriage, pre-eclampsia, premature membrane rupture, preterm labor and preterm birth (61–63). Neonates born to infected mothers presented fetal distress with abnormal respiratory gastrointestinal symptoms and refractory shock with multiple organ failure was reported (64). Furthermore, the presence of SARS-CoV-2 was detected either in amniotic fluid, umbilical cord blood or placental sections (65, 66). Vertical transmission of SARS-CoV2 has been suspected in several cases but the mechanisms that allow the virus to breach the placental fortress and reach developing fetus are still lacking. Recent analyses of infected case reports and small cohorts from infected mothers and newborn dyad, revealed the presence of SAR-CoV2 antigens or viral RNA in fetal trophoblast cells and stromal cells (65, 66). The observation of SARS-CoV-2 spike protein in both maternal and fetal side is associated with tissue necrosis and massive recruitment of inflammatory cells to the intervillous space, termed chronic histiocytic intervillositis (67). Thus, placenta from COVID-19 pregnancies are highly likely to develop tissue inflammation and abnormal or injured maternal vessels.

As a cardinal unit for growth and regeneration, fetal trophoblast cells are central for the maintenance and development of the placenta and fetus, and defense against invading pathogens. The trophoblast rich in stem cells is endowed with intrinsic defense mechanisms, which include the production of type III interferons and a set of interferon stimulated genes (ISGs). In addition, a variety of receptors known as pattern recognition receptors (PRRs) including the Toll like receptors (TLRs) are widely expressed in the fetal placenta, but, very little is known about the expression of TLRs at the maternal decidua (68). Both innate immune cells and non-immune cells of the maternal-fetal unit including decidual cells, mesenchymal and trophoblast stem cells, and the amniotic epithelium express TLRs 3, 7, 8, and 9 that can sense viral infection (69). In addition to the intrinsic defense mechanisms and inducible antiviral defenses could lead to beneficial effects by restricting viral replication and dissemination. However, the downstream signaling pathways and induced differential response may determine the final outcome. Our group and others demonstrate the placenta increases the production of a large number of ISGs including IFITM family members, IFI6 and ISG20, and type III IFNs to preempt ZIKV infection (58, 70, 71). Similarly, we reported the involvement of many factors in limiting HEV-1 infection (53). However, trophoblasts function as an active barrier preventing the transmission of certain viral infections to the fetus and sustained inflammatory response may result in disruption of the fine-tuned microenvironment leading to viral dissemination and major pregnancy disorders such as preeclampsia, or UGR.

### Immunity to Viral Infection

The “immune privilege” status of the maternal-fetal interface prevents harmful immune response to semi allogeneic embryonic cells. Nevertheless, there is an accumulating body of evidence supporting the active role of decidual immune cells in particular dNK cells and dMφ in controlling infections (72–74).

Our previous studies demonstrated that dNK cells can infiltrate HCMV-infected maternal-fetal tissues and colocalize with infected cells. dNK cells are then able to specifically detect and kill infected cells *via* the activating NKG2C/E and NKG2D receptors. They are also able to acquire specific cytotoxic functions directed against infected decidual fibroblasts (73). Interestingly, HCMV infection is also linked to a change in the secretome of dNK cells. Overall, by modulating the secretory profile of dNK cells toward increased production of cytotoxic factors, HCMV-infection likely constitutes virus-specific immune response (73).

Likewise, the secretion of IFN-γ by dNK cells during HIV-1 infection shapes the polarization of dMφ endowing them with natural resistance to infection (72, 74). This resistance is lost when the dNK cells are no longer in contact with dMφ or when the IFN-γ is neutralized by blocking antibodies, further confirming the role of the local environment in the control of the infection (72). In addition to their role during viral infection, dNK cells are able to balance the contradictory demands of fetal tolerance and protection against bacterial infection. Recent work by the group of Lieberman demonstrated that dNK cells are able to kill intracellular Listeria monocytogenes in trophoblasts and dMφ, through the transfer of granulysin cytotoxic molecules *via* tunneling nanotube (75).

The role of dMφ in the protection against pathogens did not receive much attention. However, dMφ express high levels of Toll-like receptors (TLRs 1–9) and engagement of these receptors *in vitro* enhances the secretion of pro- and anti-inflammatory cytokines that are compatible with maintenance of the fetotolerant immune environment during initiation of immune responses to pathogens (76). Likewise, dMφ antiviral innate immune response to HIV-1 is initiated by activation of TLRs 7 and 8 which in turn would restrict genome replication (74). Activation of TLR pathway induces downstream signaling cascades that ultimately lead to the production interferons (IFNs) and hundreds of IFN-stimulated genes (ISGs) with antiviral activity (77).

Our recent work highlighted the involvement of IFNs and a set of ISGs in placental defense against viral infection (53, 70).

### Cytokine Induced Immunopathology

The microenvironment of the uterine mucosa, within first semester of pregnancy, is mandatory not only for embryo implantation but also for the maternal-fetal tolerance (24). Despite the protective effect of immune cells, an overwhelming immune response at the maternal-fetal interface may constitute the other edge of the sword. Viral-induced disequilibrium of pro-inflammatory and anti-inflammatory factors could mediate tissue injury, leading to virus spread, and adverse pregnancy outcomes (78).

In the context of HCMV, modulation of the cytokine environment is considered as a potential initiator and/or exacerbator of placental and fetal injury. Indeed, HCMV infection during pregnancy is associated with a shift toward a proinflammatory cytokine status. Levels of the cytokines, TNFα, IL-1β, IL-12, and IL-17; the chemokines CCL-2 (MCP1), CCL-4 (MIP-1β), and CXCL10; and the growth factors GM-CSF and PDGF-bb were significantly elevated in amniotic fluids from congenital HCMV fetuses (79). In addition, stillborn HCMV-infected placenta have significantly elevated levels of CCL-2 and TNFα levels than uninfected placenta (80). Our team has shown that dNK cells produce high levels of sICAM-1, CXCL-1 (GROα), IL-6, Granzyme B when stimulated with infected autologous decidual cells. On the other hand, dNK cell production of CCL-4, IL-8, CXCL-10 (IP-10), GM-CSF, RANTES, and CCL-3 (MIP-1α) was significantly decreased. These changes in dNK cell secretome are necessary for the recruitment and initiation of anti-viral immune response but reduce trophoblast cell invasion and might be partially responsible for HCMV-associated fetal damages (73).

Increased production of pro-inflammatory cytokines was also associated with pregnancy disorders upon HEV-1 infection. Several pro-inflammatory factors (TNFα, IL-6, IFN-γ, and TGF-β1) were increased in the maternal-fetal interface and peripheral blood from HEV-1 infected women with adverse pregnancy outcomes (81). Our studies revealed that HEV-1 skews the cytokine, chemokine, and growth factor secretory profile in both the decidua and placenta tissues with increased levels of IL-6, sICAM-1, CCL-3, CCL-4, G-CSF, and GM-CSF (53). These alterations in the local secretome were not only correlated with viral load but also promoted tissue apoptosis and necrosis, and may be responsible for HEV-1-associated pregnancy disorders. HEV-1 infection was also associated with decrease of CXCL-10 at the maternal fetal interface that could impair the invasion/migration of the fetal trophoblast and remodeling of maternal spiral arteries, both of which are mandatory for successful placentation.

ZIKV infection has been associated modulation of many factors such as VEGF-A, MMP-2, and MMP-9 that play a role in angiogenesis and ECM degradation. In contrast, the levels of sICAM-1 and IL-8, which could favor transmission of ZIKV, were increased. We found that ZIKV does not interfere type I or type II IFNs, but induces strong increase in the production of type III IFNs (IFN-λ2/3) and a set of interferon stimulated genes (70). We also provided the first evidence that ZIKV infection induces the production of several active lipid mediators which plays can modulate the placenta pro- and anti-inflammatory responses (58). The association between ZIKV-induced pro- and anti-inflammatory lipid metabolites and cytokines/chemokines have been implicated in the physiopathology of several infectious inflammatory disorders.

Modification of the local cytokine profile has also been reported in SARS-CoV-2 infection. SARS-CoV-2 infection is associated with immune cell activation, which may be responsible for a cytokine storm, leading at least to multiple system organ failure (82). The infection is associated with increased levels of pro-inflammatory cytokines IL-6, IL-10, GCSF, CXCL10, MCP-1, MIP-1α, and TNF (83). Taken together, the cytokine storm induced during SARS-CoV-2 infection could impair the migration of trophoblasts cells and the remodeling of the maternal spiral arteries and contribute to placentation defects. The permanent increased pro-inflammatory state could also be likely to impact the fetal brain development of the fetus, thus increasing the risk of autism, mental health disorders, or schizophrenia (84).

## Concluding Remarks

Lessons from the TORCH pathogens and emerging viruses have demonstrated that the infection of the maternal-fetal interface during early pregnancy is often associated with placental diseases and is highly detrimental to fetal development. Viral tropism and efficient replication combined with altered local environment dictate the extent of tissue damage. Significant effort has been made toward understanding the essential role of dNK cells in viral spread. However, why in some cases these cells fail to control the infection or participate to tissue damage need to be further investigated. dNK cell responsiveness to pathogens can be dependent on host factors. Thus, one research axis can focus on KIR and HLA alleles expressed by the host. Indeed, several examples of matched and mismatched KIR and HLA were previously described to be protective or deleterious during viral infections. Metabolic alterations may also underlie the failure of the immune response especially that viral infections are known to deplete and/or enrich the local environment with several metabolites that alters immune cell function. Viral factors may also shape the immune response. For instance, HEV-1 and ZIKV downregulate IFNλ and other ISGs in infected target cells, favoring their own replication and spread at the maternal-fetal interface. Although, similar alterations were previously reported in cell line-based models, the mechanisms that are at play in the decidua and placenta tissue remain elusive. The impact of such mechanisms on dNK cells need also to be further investigated. Another open question is whether dNK cells can expand in response to infected cells and generate a “memory-like” response. Such a memory may generate a natural vaccine against viruses and contribute to the control of viral transmission to the fetus. Finally, a particular attention should be given to dMφ since these cells constitutes the first barrier against different infections in other tissues.

## Author Contributions

AE and NJ-F wrote and edited the manuscript. All authors contributed to the article and approved the submitted version.

## Conflict of Interest

The authors declare that the research was conducted in the absence of any commercial or financial relationships that could be construed as a potential conflict of interest.
